# Molecular Dynamics Simulation Study of the Selectivity of a Silica Polymer for Ibuprofen

**DOI:** 10.3390/ijms17071083

**Published:** 2016-07-07

**Authors:** Riccardo Concu, M. Natalia D. S. Cordeiro

**Affiliations:** REQUIMTE/Department of Chemistry and Biochemistry, Faculty of Sciences, University of Porto, Rua do Campo Alegre, 687, 4169-007 Porto, Portugal

**Keywords:** molecular dynamics, ibuprofen, molecular imprinting, xerogels, sol-gel, GROMACS, OPLS-AA

## Abstract

In the past few years, the sol-gel polycondensation technique has been increasingly employed with great success as an alternative approach to the preparation of molecularly imprinted materials (MIMs). The main aim of this study was to study, through a series of molecular dynamics (MD) simulations, the selectivity of an imprinted silica xerogel towards a new template—the (±)-2-(*P*-Isobutylphenyl) propionic acid (Ibuprofen, IBU). We have previously demonstrated the affinity of this silica xerogel toward a similar molecule. In the present study, we simulated the imprinting process occurring in a sol-gel mixture using the Optimized Potentials for Liquid Simulations-All Atom (OPLS-AA) force field, in order to evaluate the selectivity of this xerogel for a template molecule. In addition, for the first time, we have developed and verified a new parameterisation for the Ibuprofen^®^ based on the OPLS-AA framework. To evaluate the selectivity of the polymer, we have employed both the radial distribution functions, interaction energies and cluster analyses.

## 1. Introduction

Molecular imprinting technology (MIT) is a new and emerging technique based on natural molecular recognition. Using this technique, we can produce polymers with tailored recognition sites that will specifically interact with template molecules used to produce the sites. In fact, these materials are created through the interaction between the template and complementary functional monomers; this first step is then followed by the polymerisation of this conjugate with cross-linkers in an appropriate solvent. Finally, the template molecule is removed from the matrix, usually using a washing procedure, leaving the binding sites free. Due to this, MIT has been widely used in recent years in a great variety of areas to prepare the so-called molecular imprinted polymers (MIPs). These new polymers have several advantages such as physical robustness, long shelf life, simple preparation, and great selectivity. For these reasons, MITs are being studied and used in very different fields such as solid phase extraction, enantiomer separations, drug delivery, drug discovery, ligand binding assays, and to prepare synthetic receptors able to recognise and bind or release the template molecules, as well as within the new High Performance Liquid Chromatography (HPLC) matrix for selective detection and/or separation of drugs [1,2]. In particular, these polymers are extensively used in the pharmaceutical area in order to develop and produce new and highly efficient pharmaceutical devices. This is the case of new molecular imprinted nanocarriers for the sustained release of a drug [3], or new MIPs able to selectively release a drug at a specific pH [4]. Thus, MIPs have a great potential and a wide range of application.

On the other hand, sol-gel materials are obtained from the hydrolysis and polycondensation of precursors, usually with a M(OR)4 general structure, which evolves into an inorganic 3D netchapter, built up of –M–O–M– bonds. Most often, M is either Si (the gel is usually called silica) or Ti (titanium dioxide gels). R usually represents an alkyl group that does not link to the netchapter. However, it is possible to produce hybrid organic–inorganic materials by the co-polycondensation of M(OR)4 and R’-M(OR)3, where the non-reactive R’ becomes incorporated in the netchapter skeleton. Thus, it is possible to produce a large variety of gels bearing some R’ functionality. In any case, the sol-gel process can be described as follows: In an aqueous solution (in the presence of a co-solvent to prevent immiscibility), metal alkoxides (M–OR) are hydrolysed to produce M–OH groups, which will then go under condensation reactions to form a –M–O–M– network that is the foundation of the growing three-dimensional gel structure. During the time required for these reactions to take place, the viscosity of the solution gradually increases, and, when drying occurs at ambient conditions, the resultant material is denominated xerogel. Books by Brinker and Scherer [5] or Wright and Sommerdijk [6] are recommended for further reading about the physical and chemical principles of sol-gel processing.

Finally, molecular modelling stands for the general process of describing complex chemical systems in terms of a realistic model, with the goal of understanding and predicting macroscopic properties based on detailed knowledge at an atomic scale. Often, molecular modelling is used to design new materials, for which the accurate prediction of physical properties of realistic systems is required. These properties could be divided into two main groups: static equilibrium properties, like the binding constant of a drug to a receptor, and dynamic or non-equilibrium properties, like the diffusion of molecules through two phases, reaction kinetics, and so on. Due to the great variety of techniques and software, we have carefully chosen in this work the most appropriate to our problem. At a glance, there are many software packages that can perform a molecular dynamics (MD) simulation. The more famous are AMBER, CHARMM, Discovery Studio, GAUSSIAN, GROMACS, GROMOS, and LAMMPS [7,8,9,10,11,12]. Some are open source, such as GROMACS or LAMMPS, whereas others require the purchase of a licence, such as GAUSSIAN, CHARMM and Discovery Studio. GROMACS is one of the most used to perform MD simulations due to its very fast algorithmic and processor-specific optimisation, typically running 3–10 times faster than other simulation packages [13,14]. In addition, with the use of high-end Graphical Processing Unit (GPU) instead of a classical large cluster, GROMACS is even faster. Indeed, we have been able to boost our simulations up to 10 times.

**Figure 1:**
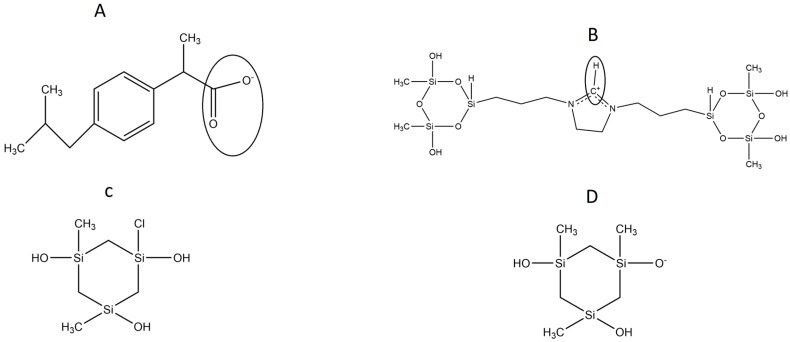
Chemical structure of the simulated molecules. **A** = Ibuprofen, IBU; **B** = Dehidroimidazolium motif modified with silica trimers, ORMOSIL, DHI^+^; **C** = Cyclic silica trimer, SI3; **D** = Anionic form of the cyclic silica trimer, SI^−^. The circles show the atoms considered in the calculation of the RDF.

In the present work, we focused our simulations on the dehydroimidazolium motif, a cationic ORMOSIL (DHI, Figure 1B), aimed at the sol-gel molecular imprinting of the carboxylate form of the non-steroidal anti-inflammatory drug (±)-2-(*P*-Isobutylphenyl) propionic acid (Ibuprofen, IBU, Figure 1A). In a previous work, we showed the selectivity of this polymer for the 2-(6-methoxynaphthalen-2-yl) propanoic acid (*S*-Naproxen, NAP). This work is focused on the IBU but we foresee a future work where the selectivity of the imprinted DHI between its template (NAP or IBU) and a similar molecule will be evaluated. Even though this is a completely new work, other authors have approached the problem of simulating the interaction between polymers and negatively charged drugs. This is the case of Bogdanova et al., who investigated the interaction between poly(vinylpyrrolidone) (PVP) and Ibuprofen and Naproxen [15]. These authors, in order to elucidate the differences in the interactions targeted, used a combination of experimental and modelling approach. Moreover, Avila-Salas investigated the interaction between polyamidoamine (PAMAM) and some nonsteroidal anti-inflammatory drug, such as difunisal, ibuprofen, ketoprofen, and naproxen [16]. More generally, the papers of Caballero et al. and Vergara-Jaque et al. are also relevant in this field [17,18].

## 2. Results

**Figure 2:**
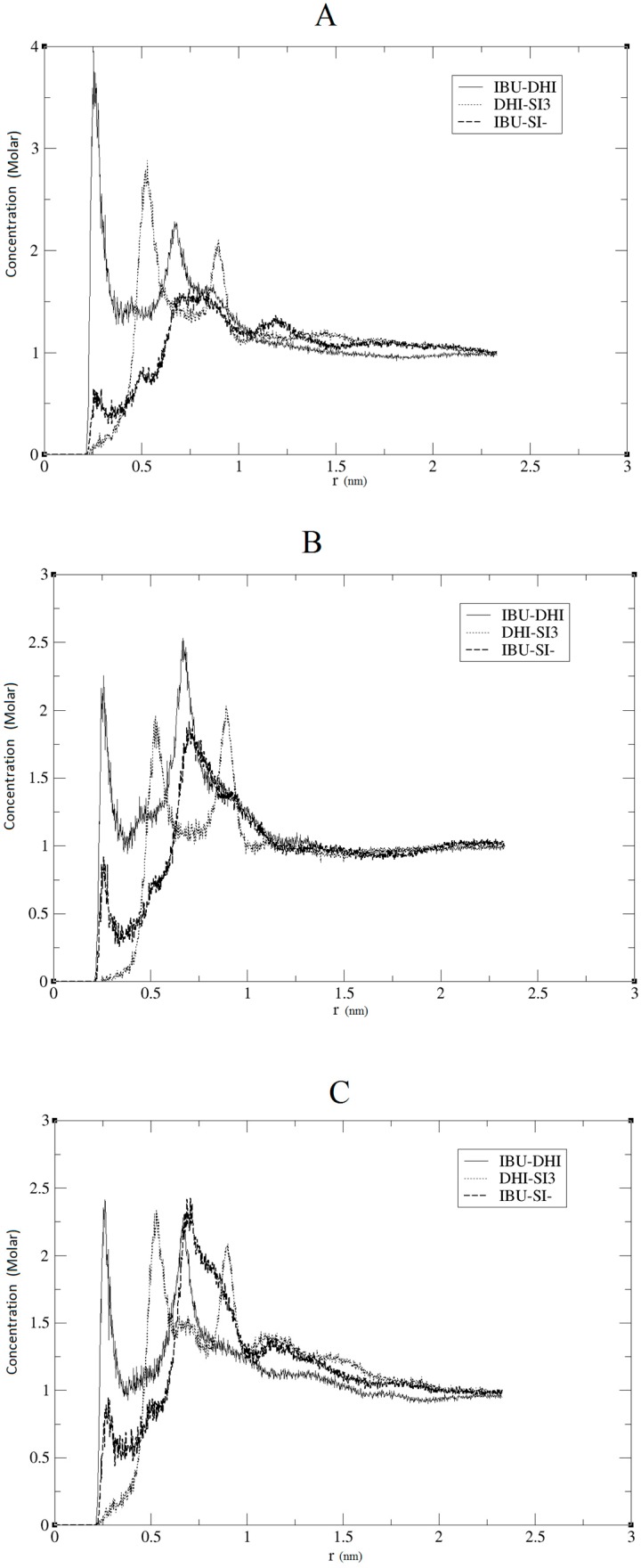
Radial distribution functions (RDF) analysis using the H atom of DHI for the pairs IBU-DHI, DHI-SI3, IBU-SI^−^. (**A**–**C**) represent the RDF of each MD replica.

**Figure 3:**
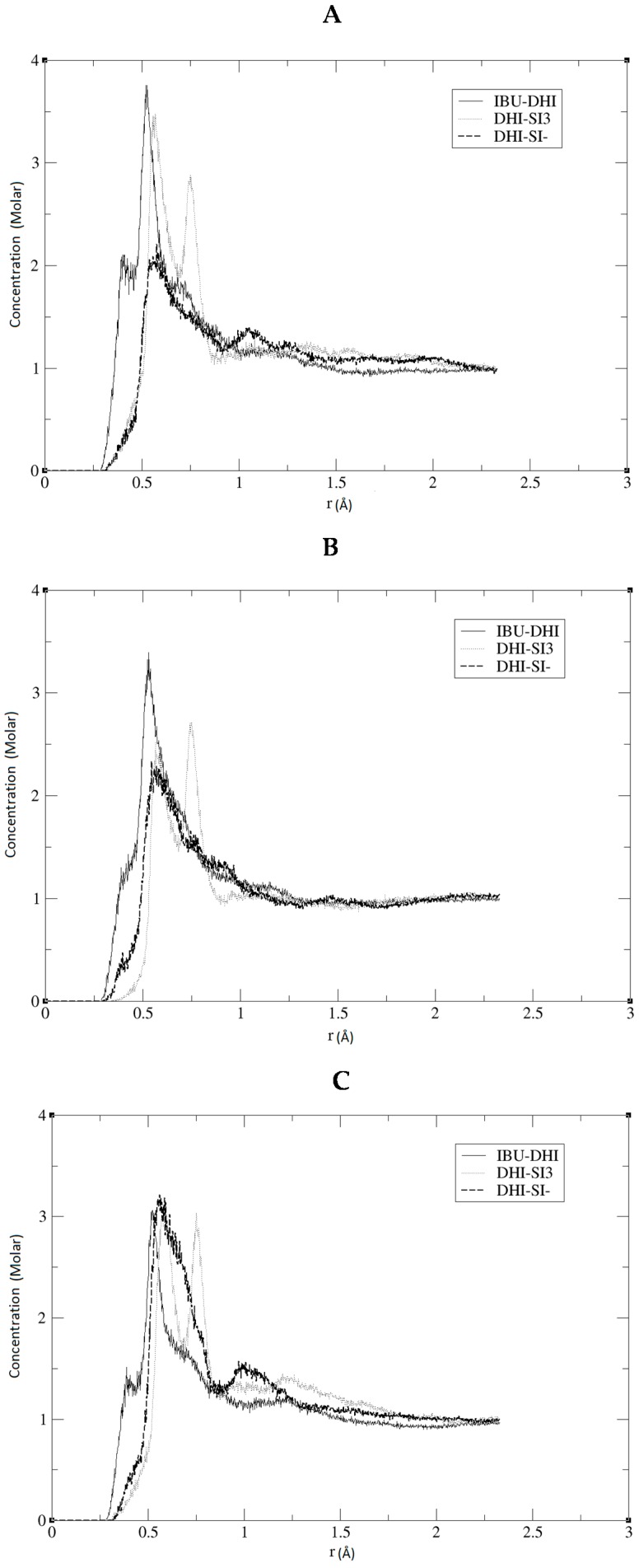
RDF analysis using the C atom of DHI for the pairs IBU-DHI, DHI-SI3, IBU-SI^−^. (**A**–**C**) represent the RDF of each MD replica.

**Figure 4:**
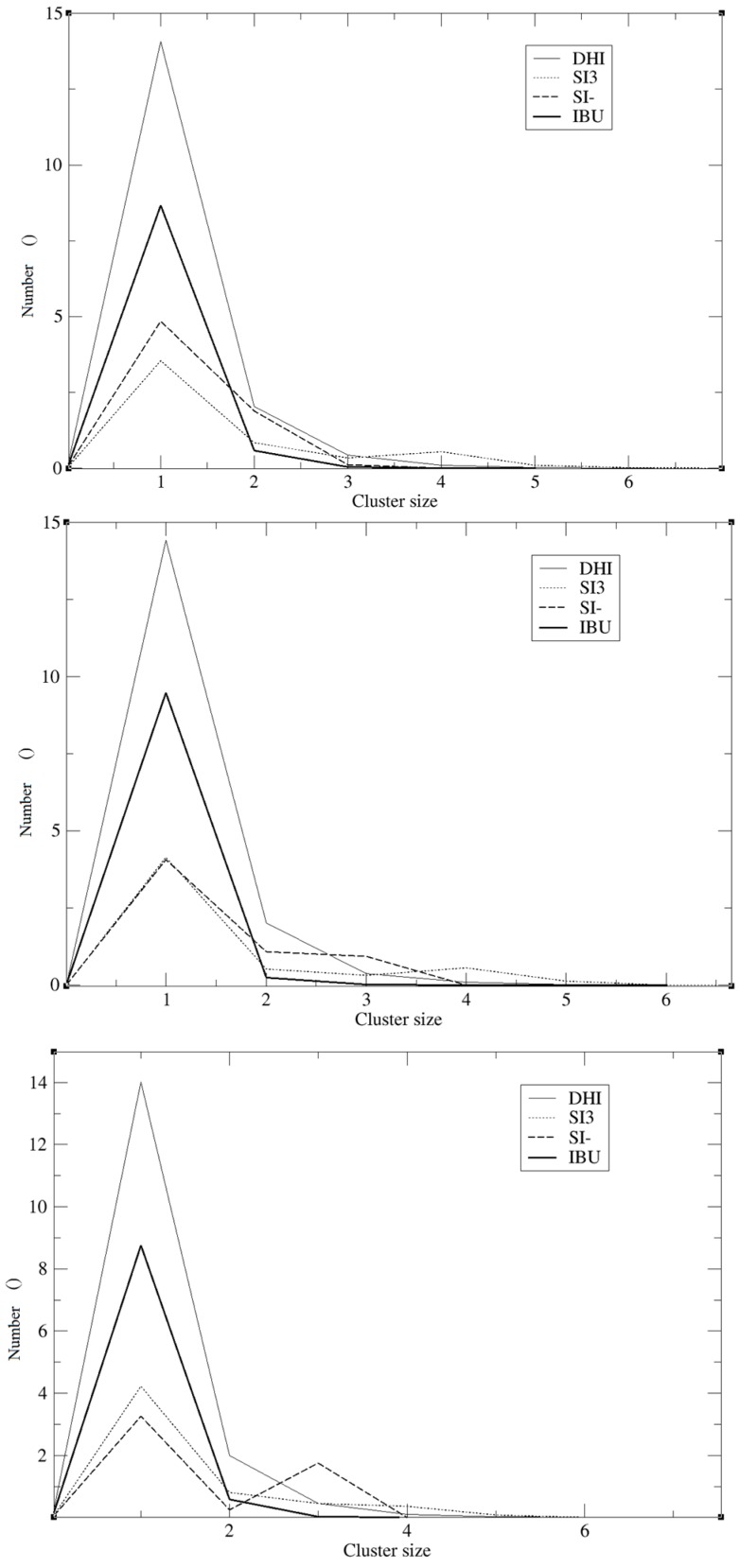
Cluster analysis of the three MD replicas.

**Figure 5:**
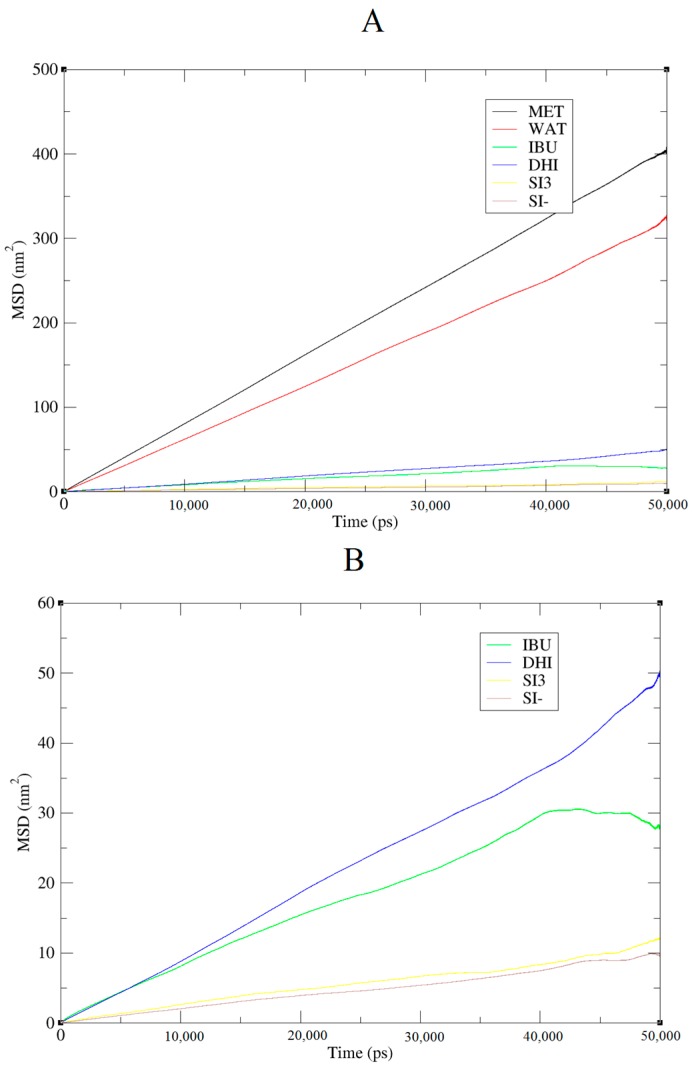
MSD of the molecular species during the simulation. (**A**) MSD of all the compounds present in the mixture; (**B**) MSD of IBU, DHI, SI3 and SI^−^.

The model herein simulated contains the DHI^+^ molecule represented in Figure 1B. We performed three independent runs, in order to ensure the robustness and the replicability of the simulations. The radial distribution functions (RDF) together with the respective running coordination numbers (*N_B_*) and cluster analyses were applied to study the affinity between the template and the polymer. The RDF were calculated using both a specific atom and the centre of the mass of the molecule. In our system, this is needed in order to evaluate the affinity of the whole molecule and the interaction between specific parts of the molecules. The atoms used for the template are the two oxygens of the carboxylic terminal, while for DHI^+^, the hydrogen and the carbon atoms were chosen in order to evaluate both the affinity and the orientation of the template when interacting with the polymer; these atoms were circle-marked in Figure 1. In Figure 2, we present the RDF calculated using the hydrogen of the DHI^+^, while, in Figure 3, we present the RDF using the C atom of the DHI^+^. The corresponding *N_B_* analysis is reported in Table 1. Regarding *N_B_*, it is important to underline that this number is calculated using the RDF first global minimum. Thus, it is strictly correlated to the distance at which the minimum is located, and a higher value does not always indicate a better affinity. In the case of the pair DHI^+^/IBU, the first is generally located at 0.5 nm, and the *N_B_* calculated is 0.24, 0.23, and 0.24. In the case of the pair DHI^+^/SI3 (cyclic silica trimer), the calculated *N_B_* values are 0.25, 0.27, and 0.28; however, in this case, the local minimum is located at 0.75–0.8 nm. In the case of the pair DHI^+^/SI^−^, the registered values are 0.005, 0.008, and 0.003, and the minimum being located at 0.5 nm. The cluster analysis presented in Figure 4 is in good agreement with the work of Pereira et al. [19], where the trend of the silica monomers to form aggregates was recorded. During the simulation, the typical cluster of the SI^−^ and SI3 is formed by three or four molecules. However, the DHI^+^ shows a further improved trend of forming very large aggregates; in fact, in this case, the cluster is usually formed by 15 molecules. In addition, the IBU forms large aggregates of eight molecules. Finally, a mean-square displacement (MSD) analysis confirmed all the other data; Figure 5 presents the diffusion of the species included in the simulation. In Figure 5A, we present a graph for all species, while, in Figure 5B, only those for DHI^+^, IBU, SI3, and SI^−^ are displayed. The MSD in this case is compatible with the data published by Li et al. [20].

## 3. Discussion

In this study, the affinity between the DHI^+^ and the Ibuprofen, the template molecule, has been simulated for the first time. The simulated system is very complex and comprises many compounds, most of them in their ionic form, for instance, IBU, DHI^+^, SI^−^, SI3, and the solvents water and methanol.

The results reported in the previous section may confirm that, in all three replicas, a good imprinting effect occurs. As can be seen in Figure 2 and Figure 3, the affinity between the template and DHI^+^ is considerably relevant in all three replicas. In fact, the RDF analysis shows that the pair IBU/DHI^+^ always has high and sharp peaks that are at shorter distances (e.g., at 0.25–0.3 nm). Another important fact is that the SI3 and SI^−^ also have a good affinity for the template. This is relevant because the affinity between the template and the monomers indicates that the simulations may reproduce the growing process of the polymer. In fact, we should take into account that the monomers, SI3 and SI^−^, are present in the simulation box because they are essential in the formation of the gel backbone. These results are further confirmed by the *N_B_* analysis where the pair IBU/DHI^+^ always has the highest number at a shorter distance, confirming the good affinity between the template and the polymer. Regarding the cluster analysis, the general trend of the species is in line with recent papers [19]. In fact, the DHI^+^ is forming a huge cluster of fifteen molecules, confirming that the simulations are able to reproduce what is really happening in a sol-gel mixture. As presented in Figure 4 the template also forms a large aggregate of seven or eight molecules. One might think that such a result may be in conflict with the RDF analysis but indeed it confirms the previous results. Indeed it is due to the proximity between the template molecules, apart from clearly showing that the IBU is embedded in the polymer. Overall, the results of this model suggest that the simulations were able to mimic the behaviour of a real system during an imprinting process. In fact, considering all these results, we can conclude that the DHI^+^ and the IBU form large aggregates and that the simulation is able to mimic a successful imprinting process as in a real system.

## 4. Material and Methods

The MD simulations were performed with the GROMACS 5.0.4 [11] package applying the OPLS-AA [21] force field, including the enhancements proposed by our group for sol-gel reagents in a recent publication [22]. GROMACS is an open source software package widely used to perform MD simulations to simulate a great variety of systems; it is one of the best programs to perform MD simulations due to its high speed and reliability—in particular, through the new GPU support, which is able to speed up a MD simulation up to 10 times [13]. All systems under study contained water, methanol, the anionic form of Ibuprofen (the template, IBU, Figure 1A), the silica trimer SI3 (Figure 1C), its anionic form SI^−^ (Figure 1D), and the dual cyclic silicate trimer corresponding to a hydrolysed and condensed species derived from the cationic dehydroimidazolium ORMOSIL (DHI^+^, Figure 1B). All these structures are depicted in Figure 1. In a previous work, we studied the DHI^+^ species with a complete –OH hydrolisation in the silica rings. In this case, we focused our investigation on testing the affinity of another ORMOSIL compound that is likely to be present in the mixture.

Due to this, we present here a new DHI^+^ species with a replacement in a 1:2 ratios in the silica rings (Figure 1C). This change was made in order to study the selectivity of the DHI^+^ molecule that is likely to be present in the real mixture. In previous works, we studied the affinity of other DHI species towards different templates. Using this kind of representation, we are able to study and simulate the affinity of two new DHI^+^ molecules for the template in order to evaluate which one has the better affinity for the template molecule. All the nonstandard parameters have been described in previous reports [22,23] except those used for the two new DHI^+^, which were parameterised and validated for the first time in this paper. With regard to the atomic point charges for the DHI^+^ and IBU species, these were calculated using GAUSSIAN 09 [10] in an OPLS-AA compliant manner, meaning that the geometry was first optimised at the HF/6-31G* level, and partial charges were then computed from a single-point run, using the CHelpG scheme [24] at the B3LYP/6-311++G(2d,2p) level of theory; information regarding these molecules is available under request. This approximation was chosen over the standard OPLS-AA force-field calculation (MP2/aug-cc-pVTZ//HF/6-31G*) due to a better stability of the DHI^+^ when using the 6-311++G(2d,2p) basis set. The studied model is specified in Table 2. The number of functional silicate (DHI^+^) units and structural silicate (SI3 plus SI^−^) units was determined from the experimental concentrations of DHI^+^ iodide and tetramethyl orthosilicate (TMOS). It was assumed that the precursors went through complete hydrolysis and fully condensed to DHI^+^ or SI3 (or its conjugated bases). On the other hand, the ratios of SI3 to SI^−^ units and DHI^+^ were estimated from a species distribution analysis conducted at pH 9, based on the acidity constant of the silanol group, bearing in mind that pKa decreases by 1–2 units with high methanol contents.

The initial state of the system was obtained using the PACKMOL package which inserts the respective number of units into the boxes at random positions [25]. Initial box dimensions were estimated considering the molecular weight and the density of each of the components of the mixture. After energy minimisation using the steepest-descent method implemented in the GROMACS package, a temperature annealing was performed in the *NVT* ensemble for 2 ns, attaining a temperature of 500 K, in order to ensure a proper mixing and gather three random independent initial configurations. These were, subsequently, used as starting configurations for the three independent MD equilibration runs needed to test the reproducibility of the simulations. Before the production stage, ~50 ns of simulation time in the *NpT* ensemble were taken to equilibrate the system and reach a stable configuration. Finally, production runs of 50 ns were performed in the *NpT* ensemble for data collection. Observable properties were sampled every 2 ps, from which total averages and standard deviations for each run were computed. The equations of motion were integrated using the Verlet leapfrog algorithm [26], with a time step of 2 fs. Typically, the temperature (*T*) was kept fixed at 298 K by applying the velocity rescaling thermostat [27], and, whenever necessary, the pressure (*p*) was held constant at 1 bar by using the Parrinello–Rahman scheme [28,29]. The time constant used for the Parrinello–Rahman coupling was set to 1 ps. Periodic boundary conditions were applied in all three Cartesian directions. For the water molecules, the Transferable Intermolecular Potential four-point model (TIP4P) [30] was applied. The non-bonded electrostatic interactions were calculated using a sixth-order particle mesh Ewald (PME) method [31] beyond a cutoff radius of 1.1 nm. The Lennard–Jones was calculated within a cutoff radius of 1.1 nm with the help of a neighbour list, updated every 10 time steps. A dielectric permittivity, ε*_r_*, equal to 1.0 was used. Statistical and trajectory analysis of the simulations were carried out by resorting to the utilities included in GROMACS, while visualisations were made with Visual Molecular Dynamics (VMD) [32]. The analysis consisted essentially in the calculation of radial distribution functions (RDF), diffusion coefficients (*D*), and coordination numbers (*N_B_*), along with clustering analyses. The RDF between different types of molecules were calculated as

(1) $$g_{AB}\left(r\right)=\frac{\langle \rho_{B}\left(r\right)\rangle }{{\langle \rho_{B}\rangle }_{loc}}$$

where $\langle \rho_{B}\left(r\right)\rangle$ refers to the average density of particle *B* at a distance *r*, around the particle *A*, and ${\langle \rho_{B}\rangle }_{loc}$ refers to the density of the particle *B* averaged over all spheres around particles *A* until a maximum radius (*r*_max_), i.e., half of the box length. The RDF are additionally averaged on all particles of type *A* present in the system and averaged over the trajectory (simulation time). The g_rdf function included in the GROMACS package calculates the RDF in different ways. The normal method is around a (set of) particle(s), the other methods are around the centre of mass of a set of particles or to the closest particle in a set. Here, the RDF were calculated using both. The NB of a particle or atom *B* around another one *A* were calculated by integrating the radial distribution function between the centre of *A* and the first local minimum, *r_m_*:

(2) $$N_{B}=4\pi \rho_{B}\int_{0}^{r_{m}}g_{AB}\left(r\right)r^{2}dr$$

where $\rho_{B}$ refers to the density of species *B* (expressed in units of molecules per volume).

The cluster analysis was performed using the g_cluster package included in the GROMACS software. This utility can cluster structures using several different methods. We determined structures from the trajectories of the runs using the single linkage, which adds a structure to a cluster when its distance to any element of the cluster is less than the cutoff. We performed the cluster analysis using cutoff values (i.e., the largest distance to be considered in a cluster) between 0.3 and 1.5 nm.

As to the diffusion coefficients of the mixture components, these were calculated from the Einstein relation (mean-square displacement (MSD)):

(3) $$D=\frac{1}{6t}{\langle |{\vec{r}}_{i}\left(t\right)-{\vec{r}}_{i}\left(0\right)|}^{2}\rangle$$

where ${\vec{r}}_{i}$ are the centre of mass positions of the molecules. The MSD is averaged over molecules, and, in order to improve the statistics, several restarts *r*(0) were used along the trajectory.

Finally, the assessment of the validity of the potential considered for the simulations was demonstrated in our previous paper [22] All the molecular species had been previously validated, save for IBU, which was parameterised for the first time here. The validation of this new molecule was performed just as in our previous work [22]. That is, a pure IBU system was considered and simulated, consisting of 100 molecules and 100 counter ions in a cubic box. The density of this template is 1.0 g/cm^3^, while the density calculated by MD was 1.084 g/cm^3^. These values appear to be acceptable according to the modifications and therefore, it allowed us to conclude that the parameterisation of the molecule is acceptable.

## Figures and Tables

**Table 1:** Coordination numbers for the most important pairs of the model.

Pair	Model A		
	1	2	3
IBU/DHI^+^	0.24	0.23	0.24
SI3/DHI^+^	0.25	0.27	0.28
SI^−^/DHI	0.005	0.008	0.003

IBU: Ibuprofen; DHI: Dehidroimidazolium motif modified with silica trimers; SI3: Cyclic silica trimer; SI^−^: Anionic form of the cyclic silica trimer.

**Table 2:** Composition of the simulated model.

	Methanol:Water Ratio	Ibuprofen	DHI	SI3	SI^−^	Water	Methanol
**Nº**	5:1	10	20	9	9	230	1130
